# Tissue-resident memory T cells orchestrate tumour-immune equilibrium

**DOI:** 10.15698/cst2019.05.187

**Published:** 2019-04-26

**Authors:** Simone L. Park, Laura K. Mackay, Jason Waithman, Thomas Gebhardt

**Affiliations:** 1 Department of Microbiology and Immunology, The University of Melbourne at the Peter Doherty Institute for Infection and Immunity, Melbourne, VIC, Australia.; 2 Telethon Kids Institute, University of Western Australia, Perth, WA, Australia.

**Keywords:** melanoma, cancer-immune equilibrium, tissue-resident memory T cells

## Abstract

The immune system can prevent tumour development by engaging in a process termed cancer immunosurveillance, during which immune cells such as T cells restrict tumour growth either by completely eradicating cancer cells in a process of ‘elimination' or by suppressing cancer cell outgrowth by establishing a state of tumour-immune ‘equilibrium'. Most cancers develop within epithelial layers of tissues but circulating T cells are largely excluded from these epithelial tissue compartments in the absence of infection or overt inflammation. In contrast, CD8^+^ tissue-resident memory T (T_RM_) cells reside permanently within epithelial layers of peripheral tissues without recirculating in blood. Accumulating evidence suggests that T_RM_ cells are found in diverse human solid cancers where they correlate with improved prognosis and can protect against tumour challenge in mice. However, the mechanisms through which these cells mediate cancer protection are poorly understood. In our recent study (Park SL et al, Nature 565(7739), 2019) we developed a melanoma model that allowed us to identify a critical role for T_RM_ cells in the establishment and maintenance of tumour-immune equilibrium in skin. Our findings provide insight into the immune cell populations important for maintaining long-term tumour dormancy in peripheral tissues and imply that targeting T_RM_ cells may serve as a novel cancer treatment strategy.

## EPICUTANEOUS MELANOMA INOCULATION: A MODEL OF TUMOUR-IMMUNE EQUILIBRIUM

**Figure 1:**
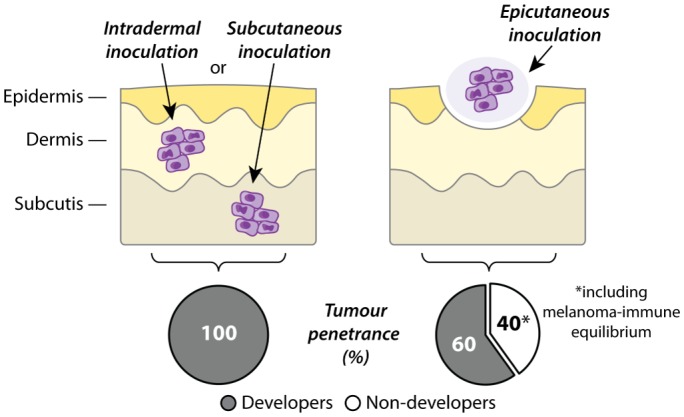
FIGURE 1: The route of inoculation determines tumour penetrance in transplantable models of cutaneous melanoma. Intradermal and subcutaneous inoculations of B16 melanoma cells (purple) result in complete tumour penetrance. By contrast, application of B16 melanoma cells onto the outermost layer of skin (epicutaneous inoculation) results in reduced tumour penetrance. Of note, the majority of non-developer mice show signs of long-term persistence of melanoma cells (melanoma-immune equilibrium).

In order to study immune responses to skin cancer, B16 melanoma cells have traditionally been grafted to mice via intradermal or subcutaneous routes, resulting in tumour growth beneath the epidermis or skin, respectively. While these models have proven highly valuable for studying immunity against rapidly progressing tumours, they nevertheless bypass the earliest developmental stages of cutaneous melanoma confined to the epidermis. In our recent study, we developed an epicutaneous melanoma model to study anti-cancer immune responses *in situ* within the uppermost layers of skin. This method of tumour inoculation initially prompts melanoma growth within the epidermis and dermis before infiltration of the subcutaneous layer and eventual metastasis to skin-draining lymph nodes, thereby approximating primary melanoma development in human patients. In contrast to the complete tumour penetrance observed after transfer of B16 melanoma cells via subcutaneous or intradermal routes, we found that only approximately 60% of mice inoculated epicutaneously went on to develop macroscopically detectable melanomas. Whereas subcutaneously inoculated tumours became uniformly palpable within one week of melanoma cell injection, epicutaneous tumours displayed far more variable growth kinetics and were usually not visible until 2-3 weeks after melanoma cell transfer. In some cases, epicutaneous melanoma growth was delayed even further and mice did not develop tumours until several weeks after inoculation. Importantly, approximately 40% of mice remained completely free of apparent melanoma disease for up to several months following epicutaneous inoculation (**Figure 1**).

**Figure 2:**
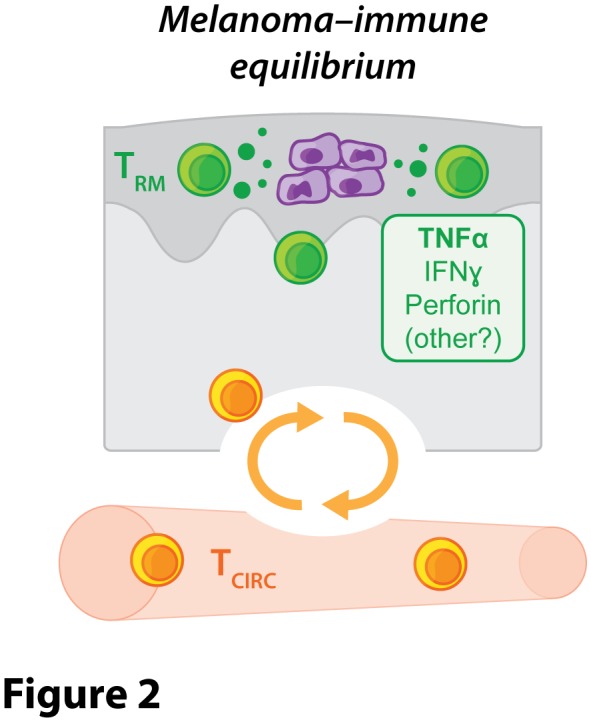
FIGURE 2: Tissue-resident memory T cells (T_RM_) suppress the outgrowth of persisting melanoma cells and promote a state of melanoma-immune equilibrium. This mode of tumour control is highly localised and does not rely on recirculating T cells (T_CIRC_). Putative effector pathways operating during melanoma-immune equilibrium include the T_RM_-derived cytokines TNFα and IFNγ, as well as the cytotoxic effector molecule perforin. While TNFα appears to play a dominant role, the contribution of the other effector molecules may be redundant.

Closer inspection of the skin of these inoculated yet apparently ‘tumour-free' mice using a variety of highly sensitive detection approaches, including bioluminescence imaging, low copy number PCR amplification or intravital 2-photon microscopy, revealed that many animals continued to harbour low numbers of viable melanoma cells in the epidermis for at least several weeks or months. These findings suggested that dormant melanoma cells could be maintained within the skin of epicutaneously inoculated mice for long periods of time without being completely eliminated (**Figure 2**). We found that the control and long-term maintenance of these dormant melanoma cells was strictly dependent upon immune cells including T cells, as mice deficient in factors required for T cell development and/or survival were highly susceptible to epicutaneous melanoma formation by comparison with wild-type mice, and ultimately failed to establish tumour dormancy. These findings suggest that B16 melanoma inoculation via the epicutaneous route might provide a unique opportunity to study the immune interactions involved in the induction and maintenance of cancer-immune equilibrium – a clinically relevant phenomenon that has typically proven difficult to study due to the paucity of murine transplantable tumour models in which tumour dormancy is observed.

## A ROLE FOR T_RM_ CELLS IN MAINTAINING TUMOUR-IMMUNE EQUILIBRIUM

To explore potential immune cell contributions to the induction and maintenance of tumour equilibrium in more depth, we used congenically marked T cells expressing a transgenic T cell receptor specific for B16 melanoma cells engineered to express a model tumour neoantigen. Adoptively transferred, tumour-specific CD8^+^ T cells were activated and recruited to the site of tumour inoculation and gave rise to T_RM_ cells expressing the canonical tissue-retentive molecules CD69 and CD103. In mice that developed progressively growing epicutaneous melanoma, tumour-specific T_RM_ cells congregated in the peritumoural skin directly adjacent to the invasive tumour margin in a similar fashion to T_RM_ cells localising to human melanomas. However, tumour-specific T_RM_ cells were far more abundant and more frequently identified in previously inoculated skin from macroscopically ‘tumour-free' mice by comparison with mice developing tumours, suggesting that anti-tumour T_RM_ cell responses can develop spontaneously in mice and correlate with improved tumour protection. These findings are aligned with the recent identification of CD103^+^ T_RM_-like cells that associate with improved patient outcomes in a variety of solid human tumours including breast, ovarian, colon and lung cancers in addition to melanoma.

By ablating memory T cells from the circulation of mice, we showed that tumour-specific T_RM_ cells were sufficient to protect against epicutaneous melanoma development in a manner that depended upon cognate antigen recognition. Rather than completely killing or eliminating cancer cells, we found that T_RM_ cells could mediate melanoma protection by upholding a state of prolonged tumour-immune equilibrium within the epidermal layer of the skin. Fluorescently tagged and tumour-specific T_RM_ cells were identified within the epidermis of macroscopically ‘tumour-free' mice where their ongoing interactions with dormant tumour cells could be visualised in real time using intravital 2-photon microscopy. Targeted depletion of T_RM_ cells from the skin of apparently ‘tumour-free' mice several weeks after melanoma inoculation catalysed tumour outgrowth in a subset of animals, indicating that tumour-specific T_RM_ cells were integral to the establishment of tumour-immune equilibrium and the maintenance of tumour dormancy. Sustainment of this cancer-immune equilibrium appeared to depend more strongly on the cytokine TNFα than on other effector molecules typically produced by CD8^+^ T cells including IFNγ or perforin, although future work needs to establish the precise contribution of these and other putatively redundant effector pathways.

In summary, our work has revealed a novel mode of anti-tumour control employed by T_RM_ cells in skin, whereby these cells can prevent tumour outgrowth without completely eliminating cancerous cells by alternatively promoting a state of tumour-immune equilibrium (**Figure 2**). This mechanism of tumour control mirrors that employed by T_RM_ cells poised to control reactivation of latent infections in barrier tissues where T_RM_ cells curb local pathogen replication to prevent symptomatic lesions as opposed to driving complete eradication. T_RM_ cells isolated from tumours can also highly express cytotoxic molecules and have been shown to kill autologous tumour cells *in vitro* but their ability to kill or eliminate tumour cells *in vivo* remains uncertain. Our findings raise the possibility that T_RM_ cells might also contribute to the initiation or control of tumour dormancy often observed in human cancer patients burdened with minimal residual disease following surgery or chemo- or immunotherapy. As such, our work suggests that enhancing T_RM_ cell responses using targeted immunotherapies might be a novel avenue through which to improve solid cancer treatments in patients.

